# Haptic-Driven Serious Card Games for Older Adults: User Preferences Study

**DOI:** 10.2196/73135

**Published:** 2025-05-27

**Authors:** Xin Huang, Nazlena Mohamad Ali, Shafrida Sahrani

**Affiliations:** 1 Institute of Visual Informatics Universiti Kebangsaan Malaysia Bangi, Selangor Malaysia

**Keywords:** serious games, older adults, haptic feedback, mobile card games, cognitive abilities

## Abstract

**Background:**

Haptic feedback technology is widely used, including in serious games. It is an additional sensory method that supplements audio and vision, provides a novel user experience through a physical control layer, and enhances the immersion of virtual reality, thereby improving the user's cognitive state while alleviating dependence on visual information. However, there is limited research on haptic feedback preferences for mobile serious card games designed for older adults.

**Objective:**

The study aims to investigate older adults' preferences for haptic feedback in mobile serious card games.

**Methods:**

This study recruited a total of 250 participants from Dangtu County, Anhui Province, China, with an average age of 64.78 (SD 4.23) years. A descriptive survey was conducted among older adults, structured questionnaires were distributed, and data were collected via the Wenjuanxing (Changsha Ranxing Information Technology Co Ltd) mobile app. Reliability and validity analyses were performed using SPSS (IBM Corp) software. The questionnaire investigated older adults' basic understanding of card games and serious games, the integration of card games with mobile devices, the potential impact of combining card games with haptic feedback technology, as well as suggestions and opinions.

**Results:**

The results showed that 63.2% (158/250) of the older adults liked the slight haptic feedback mode, and 78.4% (196/250) of the participants believed that combining haptic feedback technology with mobile card games would help improve cognitive abilities. The study found that 73.6% (184/250) of the older adults believed that this technology could reduce their reliance on visual information. This confirms that the combination of serious card games and haptic feedback can alleviate sensory impairments in older adults. Qualitative analysis revealed the potential of haptic feedback to reduce visual fatigue and provide an engaging cognitive training experience.

**Conclusions:**

Older adults have shown great interest in incorporating haptic feedback into mobile serious card games, believing that this could enhance their cognitive abilities while reducing their reliance on visual information. However, limitations include sample size and geographic restrictions, differences in digital literacy, self-reported data, and lack of longitudinal assessment. Longitudinal studies are recommended to evaluate the long-term effects of mobile serious card games with haptic feedback on cognitive abilities. Such investigations could provide valuable insights for game developers, rehabilitation institutions, and the development of cognitive training tools for older adults.

## Introduction

Touch perception can be divided into skin perception and motion perception [1], so haptic perception is composed of 2 parts of sensory feedback: haptic feedback and force and torque feedback. Devices can be classified according to the different feedback they provide. Choose the appropriate device type based on the game concept and budget estimate. The device specifications usually considered include degrees of freedom, force feedback, workspace size, maximum force and torque, stiffness, software development kit compatibility, scalability, etc. Commercial games usually prioritize affordability. A low-cost solution is to embed vibrotactile actuators into gaming devices, such as game controllers, mobile phones, etc. In general, in terms of learning and training, the force torque and haptic perception provided by haptic technology can help users better understand their performance in tasks and help them improve their performance in a more intuitive and efficient way. Especially for tasks that rely mainly on haptic feedback, pure visual feedback is of little help in improving performance and may even lead to errors. For example, without haptic feedback, endoscopic surgery training is difficult to achieve the expected results [2], the same is true for applications that focus on improving motor skills. More specifically, visually impaired users or blind people primarily benefit from auditory and haptic input. Haptic technology, as a new way to interact with virtual reality, also benefits the gaming industry in expanding its market to previously unreachable users [3].

Haptic feedback provides an additional means of haptic perception and is combined with sight and sound for a more immersive user experience. The haptic experience provides patients with a new way of perceiving, thus enhancing the user's cognitive ability. Haptic technology is a kind of sensory feedback technology that simulates haptic perception through force, vibration, or motion. It captures the user's action and interaction information through sensors and generates corresponding haptic feedback through actuators so that users can feel the real physical interaction in the virtual reality. Haptic perception is a multimodal process involving many neural pathways such as mechanoreceptors and thermoreceptors. Haptic technology simulates the haptic sensation of different materials by accurately controlling vibration frequency and intensity, thus activating multiple neural pathways and enhancing the cognitive participation of users [1]. These techniques can be used to create virtual reality objects in computer simulations, control virtual reality objects, and enhance remote control of machines and devices. Haptic feedback technology provides an immersive experience for older people by combining their sense of touch with what they see, hear, or interact with [4]. At the same time, the use of haptic technology has huge advantages, as it can provide real and intuitive interaction with virtual reality objects, improve the usability and accessibility of applications, enhance the cognitive abilities of end users, and make human-computer interaction more interesting [5]. The application of haptic feedback technology not only improves user experience but also enriches user's entertainment activities. For example, the use of haptic suits that provide haptic feedback further enhances user immersion [6].

One promising application of haptic technology is its use in enhancing cognitive function [7]. In previous studies, performing somatosensory tasks without visual input, similar to the device DOT PAD (DOT Corporation), increased pain thresholds in the trained hand while decreasing pain thresholds in the untrained hand. In addition, sensory stimulation without visual feedback resulted in changes in cortical excitability as measured by transcranial magnetic stimulation [8]. This suggests that haptic devices can modulate cortical activity and could be used to enhance cognitive function. Haptic feedback mobilizes the somatosensory system, which is closely related to cognitive functions such as attention, memory, and spatial awareness [9-11]. By providing haptic stimulation, haptic training can enhance sensory processing and promote the integration of sensory information across multiple modalities, thereby strengthening neural connections [5]. This process can improve cognitive abilities, especially in tasks that require fine motor skills, spatial reasoning, or executive function. In addition, haptic training can stimulate brain regions involved in sensory and cognitive tasks, such as the parietal cortex and prefrontal cortex, thereby promoting more coordinated and efficient neural networks [12,13]. The repetitive and goal-oriented nature of haptic tasks may also promote the development of procedural memory and enhance attentional control, further promoting cognitive improvement [14-16]. Through these mechanisms, haptic training not only facilitates the acquisition of motor skills but also provides a promising avenue for enhancing cognitive abilities, especially for people with cognitive impairment or at risk of cognitive decline.

Haptic feedback has become an integral part of serious games in recent years. A serious game is a user-participatory game that allows players to carry out activities that allow them to practice their skills and achieve goals that go beyond just enjoying leisure activities [17]. A more modern definition of a serious game is “any form of computer-based interactive game software that can be used by single or multiple players on any platform and is developed for more than just entertainment” [18]. Serious games differ from traditional video games in that they focus on problem-solving and learning rather than pure entertainment. Nonetheless, they use the same media and elements as casual video games, making them engaging and enjoyable [19]. As a serious game with a long history, easy to simulate, and relatively simple rules, card games are loved by players of all ages [20]. In addition, card games facilitate the diagnosis and tracking of cognitive impairments, reducing the burden on patients and physicians, and thus potentially providing a more comprehensive and clearer cognitive profile [21]. Card games can also be used as an effective cognitive intervention to maintain certain cognitive functions [22].

Haptic interaction can add a new dimension to the development of serious games [23]. For visually impaired users, it is difficult for them to enjoy video games. Haptic serious games offer them a whole new game experience. Yuan et al [24] discuss the accessibility of games in detail and offer insights into the challenges of future related research. Chan and Black [25] studied the performance of mental acquisition based on 3 different forms of presentation (plain text, text plus static visuals, and text plus haptic animated games). They designed experiments to teach middle school students Newtonian mechanics using each of the 3 forms of learning. The results showed that for difficult tasks, students who learned using the haptic animation serious game form performed better than other students. In addition, the user's learning experience is easier to understand than other forms. Haptic teaching helps students grasp abstract concepts more easily.

Although existing serious game research has provided valuable insights, there are still some unresolved issues. In particular, the impact of sensory decline on the interaction between serious games and older adults has not been fully explored, and there is limited research on older adults' preferences for haptic feedback in game-based training. The overall objective of this study was to explore the preference of older adults for haptic feedback in mobile card games and the potential for older adults to acquire information through haptic feedback. This study proposed the following hypotheses: first, older adults will have higher engagement and satisfaction with mobile card games with haptic enhancement. Second, older adults are more inclined to use haptic feedback to obtain information.

The rest of the paper is structured as follows. Section II outlines the research methodology, theoretical framework, and questionnaire designed based on the theoretical framework. Section III presents the research findings and details the integration of haptic feedback into a mobile card game. Section IV discusses the findings. Finally, Section V summarizes the main findings of the paper, discusses the limitations of the study, and suggests future research directions.

## Methods

### Ethical Considerations

The study was approved by the ethics committee before it was carried out and strictly adhered to the ethical guidelines for research involving human participants.

#### Research Ethics Approval

The institutional review board of the Institute of Visual Informatics, National University of Malaysia, formally reviewed and approved the study titled “Mobile Serious Gaming Technology for Enhancing Cognitive Abilities in Older Adults Using Haptic Feedback” on July 2, 2024 (approval reference: UKM.IVI.600/8/1-P136397). The study protocol, including the data collection methods, was considered to comply with the ethical standards for research involving human participants.

#### Informed Consent

Written informed consent was obtained from all participants before participating in the study. The consent form clearly outlined the purpose, procedures, risks, benefits, and voluntary nature of participating in the study. Participants were informed of their right to withdraw at any time without penalty. For secondary data analysis, the original consent form explicitly allowed the use of deidentified data for research purposes without the need for additional consent.

#### Privacy and Confidentiality

All participant data were anonymized and deidentified during collection and analysis. Personally identifiable information was removed from survey responses, and data were securely stored on a password-protected server accessible only to the research team. No identifiable information will be shared with external parties or institutions.

#### Compensation

Participants received no monetary or nonmonetary compensation for their participation in this study. Their contribution was voluntary and motivated by the potential societal benefits of the research.

#### Images and Identifiability

No images or identifiable personal information of any participant was collected or included in the manuscript or supplementary materials. All data reported were aggregated and anonymized to ensure participant confidentiality.

#### Compliance Statement

This study conformed to tenets of the Declaration of Helsinki and adhered to the ethical standards outlined by JMIR for research involving human participants.

### Research Methods Integration and Overview

This study was conducted at Dangtu Old-age University, and 250 older adults aged 60-80 years were surveyed. Among the 250 participants aged 60-80 years, 209 (83.6%) older adults had symptoms of memory loss, 3 (1.2%) older adults had mild cognitive impairment, 1 (0.4%) older adult had Alzheimer disease, and 37 (14.8%) older adults chose other. Out of 250 participants, 213 (85.2%) older adults had obvious cognitive decline. The participating older adults have basically used mobile phone software with different haptic settings. Among them, sociodemographic characteristics are usually regarded as independent variables, such as gender, age, region, work status, etc. are independent variables, and the dependent variables include participants' views on card games, acceptance of new technologies, and their views on the potential benefits of combining card games with new technologies.

The researchers evaluated vibration intensity variation and pressure-based feedback. Vibration intensity variation is suitable for fine motor skill tasks [26], while pressure-based feedback is suitable for scenarios that require precise force application [27]. The researchers ultimately chose vibration haptic feedback for the following reasons: (1) vibration feedback can provide clear and immediate haptic cues, which is crucial for maintaining user engagement and attention in cognitive tasks [28]; (2) vibration intensity can be adjusted and customized according to user preferences, and surveys show that most participants prefer mild vibration modes [29]; (3) it is technically feasible and cost-effective, making it suitable for widespread adoption [30]; and (4) vibration haptic feedback can be well applied to mobile card games [31].

The theoretical framework of this study is the Technology Acceptance Model [32] and the Multisensory Experience [33] Theory. The Technology Acceptance Model explains the user's acceptance of new technologies, including 2 key factors: perceived usefulness and perceived ease of use. The Multisensory Experience Theory emphasizes providing a rich user experience through multiple sensory channels, such as touch, vision, and hearing.

The questions in the questionnaire were designed based on the theoretical framework. The following questions are related to perceived usefulness. For example, “Do you think card games can exercise your brain?” reflects the perception of older adults on whether card games are useful in improving cognitive abilities. “Do you think card games combined with haptic feedback technology can help improve your cognitive abilities?” asks the older adults about their perception of the usefulness of haptic feedback technology in improving cognitive abilities. “What aspects of your cognitive abilities do you think card games combined with haptic feedback technology can improve?” asks older adults about their perception of the specific role of haptic feedback technology in improving cognitive abilities.

Questions related to perceived ease of use. For example, “Are you willing to try a mobile card game that reduces visual fatigue?” asks the older adults about their experience of whether mobile card games are easy to use. “Which mode of haptic feedback are you comfortable with?” asks the older adults about their comfort with haptic feedback modes and usage, which is related to perceived ease of use. Comfortable feedback modes are usually easier for users to accept and use. “Would you like to try a card game with haptic feedback technology?” asks older adults about their willingness to accept haptic feedback technology, which is often affected by their perception of the ease of use of the technology. These questions are related to perceived ease of use.

Questions related to multisensory stimulation. For example, “Which haptic feedback do you prefer?” directly involves the user's preference for haptic feedback, which is one of the multisensory experiences. “Do you think that card games incorporating haptic feedback technology could reduce the need to use vision to obtain information?” asks about the role of haptic feedback in replacing or reducing visual burden, reflecting the mutual complementation and collaboration between different senses in a multisensory experience. “Do you think card games combined with haptic feedback technology are a good combination?” asks the older adults about their overall evaluation of the combination of haptic feedback and card games, which can provide a multisensory interactive experience.

Questions related to experience richness: “Why do you like playing card games?” Understand the reasons why users like card games, which may include the multisensory experience brought by the game, such as visual patterns, auditory sound effects, and haptic operations. “What abilities of the brain do you think card games can train?” Although cognitive abilities are asked, from the perspective of multisensory experience, multisensory participation can often more comprehensively exercise the brain's multiple abilities, such as attention, memory, and thinking ability.

This study adopted a descriptive survey design [34] to investigate the preferences and opinions of older adults regarding haptic feedback in mobile card games. Data were collected through a structured questionnaire and the older adults using the Wenjuanxing [35,36] mobile app, aiming to quantitatively and qualitatively assess the respondents' acceptance of haptic-enhanced serious games. The questionnaire consisted of 4 parts: part I comprises basic cognition about card games and serious games, part II comprises a combination of card games with mobile devices, part III comprises the potential impact of combining card games with haptic feedback technology, and part IV comprises suggestions and opinions.

This study aims to quantitatively analyze and insight respondents' acceptance of new serious game designs by distributing questionnaires through the Wenjuanxing platform [35,36]. The questionnaire data were summarized as percentages and modularly analyzed using the platform to assess familiarity with card games and responses to other closed questions.

While this study provides valuable insights, certain limitations must be acknowledged. The relatively small sample size and regional focus on Dangtu County limit the generalizability of the investigation. In addition, respondents had varying levels of familiarity with digital technology, which may have influenced responses to haptic feedback.

## Results

We divided the results based on a few aspects as described below.

### Sociodemographic Characteristics

The respondents were selected by purposive sampling. A total of 250 older adults aged 60-80 years in Dangtu County were surveyed, 64 were male and 186 were female. Among them, 155 were 60-64 years old, accounting for 62% (155/250); 57 were 65-69 years old, accounting for 22.8% (57/250) of participants; 29 were 70-74 years old, accounting for 11.6% (29/250) of participants; 9 were 75-80 years old, accounting for 3.6% (9/250) of participants (Table 1).

**Table 1:** Sociodemographic characteristics of older adults aged 60-80 years in Dangtu County, China: a descriptive survey of 250 participants from October to December 2024.

Demographic characteristics		Proportion, n (%)
**Gender**		
	Male	64 (25.6)
	Female	186 (74.4)
**Age group**		
	60-64	155 (62)
	65-69	57 (22.8)
	70-74	29 (11.6)
	75-80	9 (3.6)
**Area**		
	DangTu County	250 (100)
**Work or retired**		
	Work	5 (2)
	Retired	245 (98)

### Reliability and Validity Analysis

The 14 questions in the questionnaire were analyzed for reliability and validity using SPSS data analysis software [37]. The coefficient obtained by Cronbach reliability [38] analysis was 0.729, which was between 0.7 and 0.8, indicating good reliability (refer to Table 2). The results of the validity analysis are shown in Table 3. The Kaiser-Meyer-Olkin (KMO) [39] value was 0.824, and the KMO value was greater than 0.8, indicating that the research data was very suitable for information extraction.

**Table 2:** A reliability analysis of the haptic user perception questionnaire among 250 older adults in Dangtu County, China from October to December 2024 was conducted using SPSS (Cronbach α coefficient=0.729; standardized Cronbach α coefficient=0.812).

Name	Corrected total correlation	α Coefficient with item deleted
Do you know serious games?	0.174	0.728
Are you familiar with card games?	0.307	0.719
Why do you like playing card games?	0.228	0.739
Do you think card games can exercise your brain?	0.301	0.726
What abilities of the brain do you think card games can train?	0.324	0.720
Would you like to try mobile card games?	0.414	0.712
Are you willing to try a mobile card game that reduces visual fatigue?	0.424	0.714
Which haptic feedback do you prefer?	0.402	0.714
Which mode of haptic feedback are you comfortable with?	0.438	0.703
Would you like to try a card game with haptic feedback technology?	0.520	0.706
Do you think that card games incorporating haptic feedback technology could reduce the need to use vision to obtain information?	0.520	0.707
Do you think card games combined with haptic feedback technology are a good combination?	0.557	0.705
Do you think card games combined with haptic feedback technology can help improve your cognitive abilities?	0.576	0.706
What aspects of your cognitive abilities do you think card games combined with haptic feedback technology can improve?	0.497	0.692

**Table 3:** A validity analysis^a^ of the haptic user perception questionnaire among 250 older adults in Dangtu County, China from October to December 2024 was conducted using SPSS (IBM Corp).

Name	Factor 1	Factor 2	Factor 3	Factor 4	Commonality (common factor variance)
Do you know serious games? (Factor loading coefficient)	–0.001	0.207	–0.107	0.752	0.620
Are you familiar with card games? (Factor loading coefficient)	0.183	0.018	0.147	0.742	0.606
Why do you like playing card games? (Factor loading coefficient)	0.004	0.183	0.608	–0.185	0.437
Do you think card games can exercise your brain? (Factor loading coefficient)	0.151	0.668	–0.111	0.163	0.507
What abilities of the brain do you think card games can train? (Factor loading coefficient)	0.012	0.806	0.154	0.004	0.673
Would you like to try mobile card games? (Factor loading coefficient)	0.668	0.032	–0.022	0.269	0.520
Are you willing to try a mobile card game that reduces visual fatigue? (Factor loading coefficient)	0.671	0.098	0.089	0.023	0.468
Which haptic feedback do you prefer? (Factor loading coefficient)	0.175	–0.039	0.653	0.414	0.629
Which mode of haptic feedback are you comfortable with? (Factor loading coefficient)	0.273	0.080	0.684	0.060	0.553
Would you like to try a card game with haptic feedback technology? (Factor loading coefficient)	0.843	–0.018	0.139	0.082	0.738
Do you think that card games incorporating haptic feedback technology could reduce the need to use vision to obtain information? (Factor loading coefficient)	0.792	0.073	0.140	0.074	0.657
Do you think card games combined with haptic feedback technology are a good combination? (Factor loading coefficient)	0.850	0.147	0.112	0.002	0.757
Do you think card games combined with haptic feedback technology can help improve your cognitive abilities? (Factor loading coefficient)	0.809	0.166	0.165	0.012	0.710
What aspects of your cognitive abilities do you think card games combined with haptic feedback technology can improve? (Factor loading coefficient)	0.194	0.695	0.349	0.079	0.648
Eigenvalue (before rotation) (Factor loading coefficient)	4.509	1.661	1.250	1.103	—^b^
Variance explained (before rotation; %)	32.207	11.864	8.932	7.880	—
Cumulative variance explained (before rotation; %)	32.207	44.071	53.003	60.883	—
Eigenvalue (after rotation; factor loading coefficient)	3.812	1.727	1.542	1.442	—
Variance explained (after rotation; %)	27.228	12.338	11.014	10.303	—
Cumulative variance explained (after rotation; %)	27.228	39.565	50.580	60.883	—

^a^KMO: Kaiser-Meyer-Olkin. KMO value=0.824; Bart spherical value=1161.161; *df*=91; *P*<.001.

^b^Not applicable.

### Familiarity With Card Games

In the study population, 49.6% (124/250) of the older adults were familiar with card games, with 31.2% (78/250) of those aged 60-64 years, 11.6% (29/250) of those aged 65-69 years, and further, the proportion of those aged 70-74 years familiar with card games was 6% (15/250), while for those aged 75-80 years, the proportion was 0.8% (2/250).

### Willingness to Try Mobile Card Games

Within the scope of our survey, approximately 58.8% (147/250) of older adults indicated a willingness to try mobile card games. The distribution of willingness across age groups, from highest to lowest, is as follows: those aged 60-64 years accounted for 38.8% (97/250), those aged 65-69 years accounted for 12% (30/250), those aged 70-74 years accounted for 5.6% (14/250), and those aged 75-80 years accounted for 2.4% (6/250). Concurrently, 78.4% (196/250) of older adults expressed a willingness to try mobile card games designed to alleviate visual fatigue. Among them, the proportion of those aged 60-64 years was approximately 49.6% (124/250), those aged 65-69 years was approximately 17.6% (44/250), those aged 70-74 years was approximately 8.4% (21/250), and those aged 75-80 years was approximately 2.8% (7/250).

### Motivations for Playing Card Games

In the survey regarding the reasons for enjoying card games, 23.6% (59/250) of older adults chose to play card games with friends, 43.2% (108/250) believed that playing card games could exercise their brains, 19.6% (49/250) selected playing card games to pass the leisure time, 7.2% (18/250) cited personal interest, and 6.4% (16/250) of the older adults selected other reasons (refer to Table 4).

**Table 4:** Survey results on motivation and haptic feedback preference for playing card games among 250 older adults in Dangtu County, China: A descriptive study from October to December 2024.

Survey project		Proportion (%)	Number
**Motivations for playing card games**			
	Playing with friends	23.6	—^a^
	Exercise brains	43.2	—
	Passing leisure time	19.6	—
	Personal interest	7.2	—
	Other reasons	6.4	—
**Preference for haptic feedback**			
	Haptic feedback akin to the vibration experienced when a smartphone receives a call in silent mode	30.8	77
	Uncertainty regarding the type of haptic feedback	52.4	131
	Tremor feedback, which resembles the involuntary hand tremors characteristic of Parkinson disease	9.6	24
	Shaking feedback (similar to shivering in the cold)	4.8	12
	Jiggle feedback (akin to the fine adjustments needed when attempting to open a lock with inappropriate force)	2.4	6
**Haptic feedback mode**			
	Slight haptic feedback mode, analogous to the subtle vibration experienced when a smartphone receives a text message	63.2	158
	Slightly and continuously haptic feedback mode, similar to the gentle, persistent vibration of an electric toothbrush	17.2	43
	Strong haptic feedback mode, akin to the intense vibration of a high-speed spinning washing machine	4.0	10
	Strongly and continuously haptic feedback, reminiscent of the strong and continuous vibrations felt when cycling or motorcycling over rough terrain	2.0	5

^a^Not applicable.

### Preference for Haptic Feedback

In the third section of this paper, we focus on the preferences and perceptions of older adults toward haptic feedback in mobile card games. The study revealed that 77 respondents (comprising 30.8%, 77/250 of the total sample) exhibited a preference for haptic feedback akin to the vibration experienced when a smartphone receives a call in silent mode. Regarding age distribution, 42 respondents aged between 60 and 64 years accounted for 16.8% (42/250) of the total sample; 23 respondents aged between 65 and 69 years constituted 9.2% (23/250) of the total sample; 8 respondents aged between 70 and 74 years represented 3.2% (8/250) of the total sample, and 4 respondents aged between 75 and 80 years made up 1.6% (4/250) of the total sample. Notably, 131 respondents (52.4%, 131/250 of the total sample) expressed uncertainty regarding the type of haptic feedback. In total, 24 respondents (24/250, 9.6%) of the total sample) opted for tremor feedback, which resembles the involuntary hand tremors characteristic of Parkinson disease. In addition, 12 participants chose shaking feedback (similar to shivering in the cold) and 6 participants selected jiggle feedback (akin to the fine adjustments needed when attempting to open a lock with inappropriate force), representing 4.8% (12/250) and 2.4% (6/250) of the total sample, respectively (Table 4).

### Haptic Feedback Mode

The survey findings indicate that 158 individuals (comprising 63.2%, 158/250 of the total sample) selected a slight haptic feedback mode, analogous to the subtle vibration experienced when a smartphone receives a text message. In terms of age distribution, there were 100 respondents aged 60-64 years, representing 40% (100/250) of the total sample. For those aged 65-69 years, there were 32 respondents, accounting for 12.8% (32/250) of the total sample. The 70- to 74-year age group had 21 respondents, constituting 8.4% (21/250) of the total sample, and the 75- to 80-year age group had 5 respondents, making up 2% (5/250) of the total sample. Furthermore, 43 out of 250 (17.2%) individuals opted for a slightly and continuously haptic feedback mode, similar to the gentle, persistent vibration of an electric toothbrush. In total, 10 participants (10/250, 4%) of the total sample) chose a strong haptic feedback mode, akin to the intense vibration of a high-speed spinning washing machine, while another 5 participants (5/250, 2%) of the total sample) selected a strong and continuously haptic feedback, reminiscent of the strong and continuous vibrations felt when cycling or motorcycling over rough terrain (Table 4).

### Age-Based Preferences

Overall, 40% of older adults aged 60-64 years prefer slight haptic feedback more than older adults in other age groups. This is because they are more familiar with mobile phone vibrations and have better sensory abilities than older adults in other age groups. Older adults aged 75-80 years (5/250, 2%) have a lower preference for haptic feedback, which may be due to their reduced sensitivity to haptic stimulation or greater reliance on visual information as they age.

### Haptic Feedback and Card Games Integration

The survey revealed that 163 older adult individuals (163/250, 65.2% of the total sample) expressed a willingness to try this mobile card game. Notably, within the 60- to 64-year age group, 97 out of 250 (38.8%) respondents indicated a readiness to engage with the game. For the 65- to 69-year age group, 39 out of 250 (15.6%) respondents showed their willingness to participate. In the 70- to 74-year age bracket, 19 out of 250 (7.6%) respondents expressed an interest in playing the game. Finally, within the 75- to 80-year age range, 8 out of 250 (3.2%) respondents indicated their willingness to participate in the game. Regarding the integration of haptic feedback technology with mobile card games, 187 out of 250 (74.8%) older adults deemed this combination to be an excellent pairing. In the 60- to 64-year age group, 115 out of 250 (46%) respondents considered this combination to be very favorable. Among the 65- to 69-year age group, 41 out of 250 (16.4%) respondents held this view. In the 70- to 74-year age bracket, 24 out of 250 (9.6%) respondents agreed with this combination. Finally, within the 75- to 80-year age range, 7 out of 250 (2.8%) respondents concurred with this pairing.

### Previous Gaming Experience

Older adults who had experience playing card games were more receptive to haptic feedback technology. Of the 49.6% (124/250) of older adults who were familiar with card games, 33.2% (83/250) of participants chose slight haptic feedback, indicating that familiarity with the gameplay of card games would enhance their acceptance of new technologies. This suggests that combining technology with familiar activities can improve acceptance.

### Cognitive Status

Among 250 participants aged 60-80, 83.6% (209/250) had symptoms of memory loss, 1.2% (3/250) had mild cognitive impairment, 0.4% (1/250) had Alzheimer disease, and 14.8% (37/250) chose other. In total, 85.2% (213/250) of older adults had significant cognitive decline. These data suggest that haptic feedback technology may provide them with a friendlier cognitive training experience by reducing reliance on visual information.

### Enhancing Cognitive Abilities

This study observed that 196 older adult individuals (comprising 78.4%, 196/250 of the sample) believed that the combination of haptic feedback technology with mobile card games is beneficial for enhancing cognitive abilities, a viewpoint that was uniformly recognized across all age groups. Notably, among respondents aged 75- to 80-year, 8 out of 250 (3.2%) individuals held this opinion. Following closely, 25 out of 250 (10%) respondents aged 70-74 years agreed with this combination, and 40 out of 250 (16%) respondents aged 65-69 years also believed that this integration could improve cognitive functions. Furthermore, 123 out of 250 (49.2%) respondents aged 60-64 years concurred that this combination could enhance cognitive performance.

### Reducing Reliance on Visual Information

In the exploration of older adult individuals' perceptions toward mobile card games integrated with haptic feedback technology, our study found that 184 out of 250 (73.6%) older adult participants believed that this technology could reduce their dependence on visual information. Among them, 115 individuals aged 60-64 years, accounting for 46% (115/250) of the respondents in that age group, held this view. In the 65- to 69-year age group, 39 participants, representing 15.6% (39/250) of the respondents in that age group, shared this perspective. Within the 70- to 74-year age bracket, 22 older adult individuals agreed with this combination, constituting 8.8% (22/250) of the total sample. Finally, in the 75- to 80-year age group, 8 older adult individuals concurred with this combination, representing 3.2% (8/250) of the total sample.

### Technological Integration

In the survey, the positive views of older adults individuals regarding the integration of haptic feedback technology in mobile card games for enhancing cognitive abilities were evident, with specific findings as follows: 108 older adults individuals (approximately 43.2%, 108/250) believed that the technology could improve their reaction capabilities, 66 older adults individuals (approximately 26.4%, 66/250) thought it could enhance their memory, and 47 older adults individuals (approximately 18.8%, 47/250) felt that it could improve their thinking abilities.

### Long-Term Engagement and Effectiveness

Although this study focused on initial user preference, it is critical to evaluate the long-term engagement and effectiveness of haptic feedback technology in serious games. Qualitative analysis showed that older adults believed that haptic feedback made games attractive and could promote continued engagement. Haptic feedback technology provides another way to conduct long-term cognitive training. At the same time, serious card games incorporating haptic feedback can reduce visual dependence and may lead to sustained improvements in cognitive areas such as memory, attention, and problem-solving ability.

### Opinions and Suggestions

In the final stage of the study, since this was not a mandatory question, the research team collected qualitative feedback from 31 older adult respondents to investigate their opinions and suggestions on incorporating haptic feedback technology into mobile card games. We categorized the qualitative responses into 5 distinct groups. The first category of participants emphasized the importance of regular engagement in card games for maintaining cognitive function, highlighting the need for such activities to “keep the mind sharp.” Another group expressed a willingness to explore methods that could enhance memory and improve their lifestyle, indicating an openness to new cognitive tools. A third group considered that serious games could enrich the daily lives of older adults, making their experiences “more colorful and enjoyable.” Furthermore, there was enthusiasm for the integration of haptic technology, with the fourth category of participants noting their excitement about the potential fun it could bring. The final group of participants expressed a belief that these games have the potential to enhance memory and may help prevent dementia.

## Discussion

### Visual Accessibility and Multisensory Engagement

Card games have significant health benefits, particularly in improving sleep quality. A study of 4718 older adults showed that 62% (155/250) reported good sleep quality, and those who played Mahjong daily were more likely to report better sleep quality than those who did not play Mahjong [40]. The integration of new technologies, such as artificial intelligence and haptic feedback, has further enhanced the appeal of card games to older adults [41]. The multiplayer card game Dou Di Zhu [42] remains the most popular game in China with its optimal player characters, deck composition, and scoring system [43]. Innovations such as haptic feedback in mobile card games provide a more realistic experience, reduce visual fatigue, and encourage older players to adopt digital platforms. In virtual reality [44], immersive experiences such as CardsVR promote social interaction and provide realistic haptic sensations, significantly enhancing presence indicators such as action possibilities, realism, and touch [45].

### User Preference for Haptic Feedback Intensity

In-depth interviews revealed that older adults were particularly receptive to vibration-based haptic feedback. Their familiarity with mobile phone vibrations (eg, call alerts and message notifications) also contributed to this preference. In addition, slight haptic feedback was perceived as comfortable and beneficial for relieving muscle stiffness and addressing age-related muscle and joint mobility decline [46]. These findings highlight the adaptability of older adults to new technologies when they combine them with familiar elements.

### User Preferences (Immersive Benefit and Cognitive Effect)

Haptic feedback technology also enhances cognitive engagement in card games, offering great potential for cognitive intervention. Card games combined with haptic sensations simulate real-world interactions and increase realism and immersion. This innovation is consistent with research highlighting the role of card games in maintaining cognitive function and tracking cognitive impairment [47-49]. In addition, the slight haptic feedback in the haptic feedback can further support cognitive health by reducing physical discomfort and promoting sustained engagement.

The analysis of the research results based on the theoretical framework shows that 78.4% (196/250) of older adults believe that mobile card games combined with haptic feedback can improve their cognitive abilities. This shows that older adults have a high perceived usefulness of haptic feedback technology. Similarly, 63.2% (158/250) of the older adults prefer a slight haptic feedback mode, which shows that the older adults believe that this haptic feedback mode is more suitable for the older adults and that the older adults feel comfortable. In total, 73.6% (184/250) of the older adults believe that haptic feedback technology can reduce dependence on visual information, which shows that multisensory experience can reduce visual fatigue while enhancing user immersion and engagement. Overall, 74.8% (187/250) of the older adults believe that haptic feedback technology combined with card games is a good combination, which shows that multisensory experience can enhance user experience.

### Design Recommendations

Older adults of all ages rated the combination of haptic feedback and mobile card games as a beneficial innovation. These technologies improve the gaming experience for visually impaired users [50] and help alleviate vision-related challenges associated with aging. By providing an alternative way to receive game information, haptic feedback enhances accessibility and cognitive stimulation. Overall, these innovations suggest that mobile card games with haptic feedback can have a positive impact on cognitive function and overall quality of life in older adults by promoting engagement and enjoyment.

Future serious games should provide rich sensory experiences through multimodal stimulation design to enhance user engagement and cognitive effects. The intensity and mode of haptic feedback should be personalized according to the haptic sensitivity and cognitive ability of older adults to ensure that each older person can obtain the best user experience and cognitive effects. In addition, game design should have tasks with progressive difficulty to ensure the challenge of the game, which can also be achieved by dynamically adjusting haptic feedback.

When designing haptic feedback technology, the accessibility and inclusiveness of the technology should be ensured, especially for older adults with sensory impairments. Overall, 73.6% (184/250) of older adults believe that haptic feedback technology can reduce dependence on visual information, which shows the importance of inclusive design. At the same time, the security and privacy protection of user data are also key to technology design. Technology design should also avoid exacerbating digital barriers for older adults and ensure that older adults with different digital literacy levels can use it.

The results provide valuable insights for game developers, indicating that slight haptic feedback is more popular with older adults. At the same time, the design of haptic technology should focus on multisensory experience to enhance user immersion and engagement. Based on the haptic preferences of older adults when using mobile card games, the researchers will design a serious mobile card game that includes slight haptic feedback and is suitable for older adults. This serious card game can improve older adults' cognition while alleviating their visual impairment.

This study extends the existing literature by focusing on older adults’ preferences for haptic feedback in serious card games, an area that remains underexplored. Previous studies have highlighted the potential of serious games to improve cognitive abilities, but often overlook the specific needs and preferences of older adults [51]. Our findings are consistent with these studies, demonstrating the positive response of interactive technologies on cognitive function [52]. However, this study uniquely identifies the potential of haptic feedback in reducing visual dependence in older adults, a new contribution to the field.

In addition, the high acceptance rate of haptic feedback technology by participants supports previous findings that older adults are more likely to accept new technologies when they are combined with familiar activities [53]. This study also builds on the research of Abd-Alrazaq et al [54]. They report that serious games can improve cognitive abilities in older adults with mild cognitive impairment, who experience memory loss as they age [55]. Our results suggest that haptic feedback technology can further enhance these benefits by providing a more immersive and accessible gaming experience.

### Limitations

The limitations of this study are mainly reflected in sample size and geographical restrictions, differences in digital literacy, self-reported data, and lack of longitudinal evaluation. First, the sample size was limited to 250 older adults in Dangtu County, Anhui Province, China, which may affect the generalizability of the research results. Future studies should expand the sample size and research area. Second, the digital literacy of the participants varied, which may affect their response to haptic feedback, thereby limiting the ability of the research results to be generalized to different digital literacy populations. Third, this study relied on self-reported data, and future studies could supplement it with objective measurement methods such as cognitive performance tests and usability evaluations. Finally, this study did not evaluate the long-term effects of haptic feedback on cognitive ability in serious games. It is recommended that future longitudinal studies be conducted to evaluate its continued impact.

### Future Directions

Haptic feedback technology has potential application value in cognitive training. Providing haptic stimulation can help older adults obtain more information, thereby promoting the improvement of cognitive function. The focus of future work should be to explore adaptive haptic feedback models based on user progress and cognitive response. Such a model can dynamically adjust the intensity, frequency, and mode of haptic feedback in a timely manner according to the users' real-time performance and cognitive state, providing personalized and more effective feedback to achieve better services for older adults.

### Conclusions

This study explored the haptic preferences of older adults for haptic feedback integrated into a mobile serious card game. The results showed that older adults showed strong interest in this combination, believing it could improve cognitive abilities while reducing reliance on visual information. These findings highlight older adults' preference for haptic feedback in such games.

## Abbreviations

KMO: Kaiser-Meyer-Olkin

## Data Availability

The datasets generated or analyzed during this study are available from the corresponding author on reasonable request.
